# Whole-Genome Draft Sequences of Six Commensal Fecal and Six Mastitis-Associated *Escherichia coli* Strains of Bovine Origin

**DOI:** 10.1128/genomeA.00753-16

**Published:** 2016-07-28

**Authors:** Andreas Leimbach, Anja Poehlein, Anika Witten, Olga Wellnitz, Nahum Shpigel, Wolfram Petzl, Holm Zerbe, Rolf Daniel, Ulrich Dobrindt

**Affiliations:** a Institute for Hygiene, University of Münster, Münster, Germany; b Department of Genomic and Applied Microbiology, Göttingen Genomics Laboratory, Institute of Microbiology and Genetics, Georg-August-University of Göttingen, Göttingen, Germany; c Institute for Molecular Infection Biology, Julius-Maximilians-University of Würzburg, Würzburg, Germany; d Institute for Human Genetics, University of Münster, Münster, Germany; e Veterinary Physiology, Vetsuisse Faculty University of Bern, Bern, Switzerland; f Koret School of Veterinary Medicine, The Hebrew University of Jerusalem, Jerusalem, Israel; g Clinic for Ruminants, Ludwig-Maximilians-University of Munich, Oberschleißheim, Germany

## Abstract

The bovine gastrointestinal tract is a natural reservoir for commensal and pathogenic *Escherichia coli* strains with the ability to cause mastitis. Here, we report the whole-genome sequences of six *E. coli* isolates from acute mastitis cases and six *E. coli* isolates from the feces of udder-healthy cows.

## GENOME ANNOUNCEMENT

Although bovine intramammary infections with *Escherichia coli* mostly lead to an acute onset of mastitis, they can also result in a persistent infection of the udder with alternating subclinical or clinical periods (1). Additionally, no common virulence factor subset of mastitis-causing *E. coli* strains has been identified in previous studies (2).

To investigate the genomic potential of *E. coli* isolated from bovine mastitis, several draft genomes (3–5), as well as two complete genomes (6), have been published thus far. However, only two recent genomic *E. coli* mastitis studies included one commensal bovine isolate (7, 8). Because cows are a natural reservoir not only for pathogenic but also for commensal *E. coli* of high phylogenetic and genotypic diversity (2), we present here the draft genomes of six *E. coli* strains isolated from serous udder exudate of mastitis-afflicted cows and six *E. coli* strains isolated from the feces of udder-healthy cows (Table 1).

All genomes were sequenced with an Illumina HiScan SQ sequencer with Nextera XT chemistry (Illumina, San Diego, CA, USA) for library preparation and a 101-bp paired-end sequencing run. Raw reads were quality controlled with FastQC version 0.11.2 (http://www.bioinformatics.bbsrc.ac.uk/projects/fastqc). Low-quality reads and adapter contaminations were trimmed with Cutadapt version 1.6 (9). All reads were randomly subsampled to an approximate 70-fold coverage for each strain with seqtk version 1.0-r32 (https://github.com/lh3/seqtk). Subsequently, the reads were *de novo* assembled with SPAdes version 3.1.1 (10). Assembly statistics were evaluated with QUAST version 2.3 (11), resulting in 59 to 290 contigs >500 bp and genome sizes ranging from 4,765,494 to 5,459,392 bp (Table 1).

The strains were classified evenly into phylogroups A or B1, regardless of isolation source, through the assignment of sequence types (ST) with e. coli_mlst version 0.3 (https://github.com/aleimba/bac-genomics-scripts/tree/master/ecoli_mlst) (12). The most prominent sequence type is ST10, but most of the strains were not closely phylogenetically related.

All genomes were annotated with Prokka version 1.9 (13) with *E. coli* 1303 (CP009166 to CP009169) or *E. coli* ECC-1470 (CP010344 to CP010345) as references for annotation for either the ECOR phylogroup A or B1 genomes, respectively. tRNAs were predicted with tRNAscan-SE version 1.3.1 (14). Additionally, the annotations were manually curated with Proteinortho version 5.11 (15), po2anno version 0.2 (https://github.com/aleimba/bac-genomics-scripts/tree/master/po2anno), ACT version 13.0.0 (16), and *E. coli* strains 1303 and ECC-1470 as references. Finally, tbl2tab version 0.2 (https://github.com/aleimba/bac-genomics-scripts/tree/master/tbl2tab) and Artemis version 16.0.0 (17) were used to refine the annotations after querying the Virulence Factors Database (18) and the ResFinder version 2.1 (19), VirulenceFinder version 1.2 (20), and SerotypeFinder version 1.0 (21) databases. In summary, between 4,350 and 5,211 coding DNA sequences were identified in the genomes with 3 to 7 rRNAs and 68 to 83 tRNAs.

The genome sequences in this study will serve as a useful resource for future comparative studies of *E. coli* strains associated with bovine mastitis in relationship to commensal strains and for the identification of potential virulence factors.

### Nucleotide sequence accession numbers.

These whole-genome shotgun projects have been deposited at DDBJ/EMBL/GenBank under the accession numbers listed in Table 1. The versions described here are the first versions.

**TABLE 1:** Genome features and assembly metrics of the 12 *E. coli* whole-genome sequences

Strain	ECOR phylogroup (ST)	Source of isolation	Genome size (bp)	No. of contigs >500 bp	*N*_50_ (bp)	No. of CDSs^*a*^	Accession no.
131/07	A (ST10)	Udder acute mastitis	5,459,392	270	79,414	5,123	JXUH00000000
2772a	B1 (ST156)	Udder acute mastitis	4,949,901	93	163,837	4,621	LCVG00000000
3234/A	A (ST10)	Udder acute mastitis	5,482,981	290	95,923	5,211	LCVH00000000
MPEC4839	A (ST10)	Udder acute mastitis	4,866,885	124	133,521	4,502	JYHP00000000
MPEC4969	B1 (ST1125)	Udder acute mastitis	4,833,611	130	103,834	4,468	JYHQ00000000
RiKo 2299/09	B1 (ST448)	Healthy cow feces	4,954,750	129	114,991	4,587	JYKB00000000
RiKo 2305/09	B1 (ST410)	Healthy cow feces	4,806,931	123	129,952	4,429	JYPB00000000
RiKo 2308/09	A (ST167)	Healthy cow feces	5,112,873	186	83,735	4,685	LCVI00000000
RiKo 2331/09	B1 (ST1614)	Healthy cow feces	4,765,494	59	224,192	4,350	LCVJ00000000
RiKo 2340/09	A (ST167)	Healthy cow feces	5,024,854	204	82,522	4,568	LAGW00000000
RiKo 2351/09	B1 (ST88)	Healthy cow feces	5,297,190	252	102,610	4,931	LAUC00000000
UVM2	A (ST1091)	Udder acute mastitis	4,926,170	149	86,033	4,614	LAUD00000000

a CDS, coding sequence.

## References

[1] Zadoks RN, Middleton JR, McDougall S, Katholm J, Schukken YH 2011 Molecular epidemiology of mastitis pathogens of dairy cattle and comparative relevance to humans. J Mammary Gland Biol Neoplasia 16:357–372. doi:10.1007/s10911-011-9236-y. 21968538 PMC3208832

[2] Blum SE, Leitner G 2013 Genotyping and virulence factors assessment of bovine mastitis *Escherichia coli*. Vet Microbiol 163:305–312. doi:10.1016/j.vetmic.2012.12.037. 23374653

[3] Blum S, Sela N, Heller ED, Sela S, Leitner G 2012 Genome analysis of bovine-mastitis-associated *Escherichia coli* O32:H37 strain P4. J Bacteriol 194:3732. doi:10.1128/JB.00535-12. 22740662 PMC3393488

[4] Kempf F, Loux V, Germon P 2015 Genome sequences of two bovine mastitis-causing *Escherichia coli* strains. Genome Announc 3(2):e00259-15. doi:10.1128/genomeA.00259-15. 25858841 PMC4392153

[5] Richards VP, Lefébure T, Pavinski Bitar PD, Dogan B, Simpson KW, Schukken YH, Stanhope MJ 2015 Genome based phylogeny and comparative genomic analysis of intra-mammary pathogenic *Escherichia coli*. PLoS One 10:e0119799. doi:10.1371/journal.pone.0119799. 25807497 PMC4373696

[6] Leimbach A, Poehlein A, Witten A, Scheutz F, Schukken Y, Daniel R, Dobrindt U 2015 Complete genome sequences of *Escherichia coli* strains 1303 and ECC-1470 isolated from bovine mastitis. Genome Announc 3(2):e00182-15. doi:10.1128/genomeA.00182-15. 25814601 PMC4384141

[7] Blum SE, Heller ED, Sela S, Elad D, Edery N, Leitner G 2015 Genomic and phenomic study of mammary pathogenic *Escherichia coli*. PLoS One 10:e0136387. doi:10.1371/journal.pone.0136387. 26327312 PMC4556653

[8] Kempf F, Slugocki C, Blum SE, Leitner G, Germon P 2016 Genomic comparative study of bovine mastitis *Escherichia coli*. PLoS One 11:e0147954. doi:10.1371/journal.pone.0147954. 26809117 PMC4725725

[9] Martin M 2011 Cutadapt removes adapter sequences from high-throughput sequencing reads. EMBnetJ 17:10. doi:10.14806/ej.17.1.200.

[10] Bankevich A, Nurk S, Antipov D, Gurevich AA, Dvorkin M, Kulikov AS, Lesin VM, Nikolenko SI, Pham S, Prjibelski AD, Pyshkin AV, Sirotkin AV, Vyahhi N, Tesler G, Alekseyev MA, Pevzner PA 2012 SPAdes: a new genome assembly algorithm and its applications to single-cell sequencing. J Comput Biol 19:455–477. doi:10.1089/cmb.2012.0021. 22506599 PMC3342519

[11] Gurevich A, Saveliev V, Vyahhi N, Tesler G 2013 QUAST: quality assessment tool for genome assemblies. Bioinformatics 29:1072–1075. doi:10.1093/bioinformatics/btt086. 23422339 PMC3624806

[12] Wirth T, Falush D, Lan R, Colles F, Mensa P, Wieler LH, Karch H, Reeves PR, Maiden MC, Ochman H, Achtman M 2006 Sex and virulence in *Escherichia coli*: an evolutionary perspective. Mol Microbiol 60:1136–1151. doi:10.1111/j.1365-2958.2006.05172.x. 16689791 PMC1557465

[13] Seemann T 2014 Prokka: rapid prokaryotic genome annotation. Bioinformatics 30:2068–2069. doi:10.1093/bioinformatics/btu153. 24642063

[14] Lowe TM, Eddy SR 1997 tRNAscan-SE: a program for improved detection of transfer RNA genes in genomic sequence. Nucleic Acids Res 25:955–964. doi:10.1093/nar/25.5.0955. 9023104 PMC146525

[15] Lechner M, Findeiss S, Steiner L, Marz M, Stadler PF, Prohaska SJ 2011 Proteinortho: detection of (co-)orthologs in large-scale analysis. BMC Bioinformatics 12:124. doi:10.1186/1471-2105-12-124. 21526987 PMC3114741

[16] Carver TJ, Rutherford KM, Berriman M, Rajandream MA, Barrell BG, Parkhill J 2005 ACT: the Artemis comparison tool. Bioinformatics 21:3422–3423. doi:10.1093/bioinformatics/bti553. 15976072

[17] Rutherford K, Parkhill J, Crook J, Horsnell T, Rice P, Rajandream MA, Barrell B 2000 Artemis: sequence visualization and annotation. Bioinformatics 16:944–945. doi:10.1093/bioinformatics/16.10.944. 11120685

[18] Chen L, Xiong Z, Sun L, Yang J, Jin Q 2012 VFDB 2012 update: toward the genetic diversity and molecular evolution of bacterial virulence factors. Nucleic Acids Res 40:D641–D645. doi:10.1093/nar/gkr989. 22067448 PMC3245122

[19] Zankari E, Hasman H, Cosentino S, Vestergaard M, Rasmussen S, Lund O, Aarestrup FM, Larsen MV 2012 Identification of acquired antimicrobial resistance genes. J Antimicrob Chemother 67:2640–2644. doi:10.1093/jac/dks261. 22782487 PMC3468078

[20] Joensen KG, Scheutz F, Lund O, Hasman H, Kaas RS, Nielsen EM, Aarestrup FM 2014 Real-time whole-genome sequencing for routine typing, surveillance, and outbreak detection of verotoxigenic *Escherichia coli*. J Clin Microbiol 52:1501–1510. doi:10.1128/JCM.03617-13. 24574290 PMC3993690

[21] Joensen KG, Tetzschner AM, Iguchi A, Aarestrup FM, Scheutz F 2015 Rapid and easy *in silico* serotyping of *Escherichia coli* isolates by use of whole-genome sequencing data. J Clin Microbiol 53:2410–2426 doi:10.1128/JCM.00008-15. 25972421 PMC4508402

